# Roles of DANCR/microRNA-518a-3p/MDMA ceRNA network in the growth and malignant behaviors of colon cancer cells

**DOI:** 10.1186/s12885-020-06856-8

**Published:** 2020-05-18

**Authors:** Yi Sun, Bin Cao, Jingzhen Zhou

**Affiliations:** 1 Department of Clinical Laboratory, HwaMei Hospital; Ningbo Institute of Life and Health Industry, University of Chinese Academy of Sciences; Key Laboratory of Diagnosis and Treatment of Digestive System Tumors of Zhejiang Province, No.41 Northwest Street, Ningbo, 315000 Zhejiang, People’s Republic of China; 2 Department of Clinical Laboratory, Yunlong Health Center, Ningbo, 315000 Zhejiang, People’s Republic of China

**Keywords:** Colon Cancer, Long noncoding RNA DANCR, MicroRNA-518a-3p, Murine double minute 2, Competing endogenous RNA

## Abstract

**Background:**

The competing endogenous RNA (ceRNA) networks of long non-coding RNAs (lncRNAs) and microRNAs (miRs) have aroused wide concerns. The study aims to investigate the roles of lncRNA DANCR-associated ceRNA network in the growth and behaviors of colon cancer (CC) cells.

**Methods:**

Differentially expressed lncRNAs between CC and paracancerous tissues were analyzed using microarrays and RT-qPCR. Follow-up studies were conducted to evaluate the correlation between DANCR expression and prognosis of CC patients. Loss-of-functions of DANCR were performed to identify its role in the malignant behaviors of CC cells. Sub-cellular localization of DANCR and the potential targets of DANCR were predicted and validated. Cells with inhibited DANCR were implanted into nude mice to evaluate the tumor formation and metastasis in vivo.

**Results:**

DANCR was highly-expressed in CC tissues and cell lines, and higher levels of DANCR were linked with worse prognosis and less survival time of CC patients. Silencing of DANCR inhibited proliferation, viability, metastasis and resistance to death of CC cells. DANCR was found to be sub-localized in cytoplasmic matrix and to mediate murine double minute 2 (MDM2) expression through sponging miR-518a-3p in CC cells, during which the Smad2/3 signaling was activated. Likewise, silencing of DANCR in CC cells inhibited tumor formation and metastasis in vivo.

**Conclusion:**

This study provided evidence that silencing of DANCR might inhibit the growth and metastasis of CC cells through the DANCR/miR-518a-3p/MDM2 ceRNA network and the defect of Smad2/3 while activation of the p53 signaling pathways. This study may offer novel insights in CC treatment.

## Background

Colon cancer (CC), or colorectal cancer (CRC), is a cancer type that initiates from large bowel [1]. CC is one of the most commonly diagnosed malignancies in humans and results in mortality worldwide, which accounts for 600,000 cancer-caused deaths around the world each year [2, 3]. The causes of CC are considered to be linked to both genetic factors and lifestyles, such as smoking, aging, diet, and obesity [4]. Primary therapy for CC is segmental or total colectomy followed by an anastomosis, and when necessary, adjuvant chemotherapy may need to be introduced [3]. Despite the currently improved therapeutic strategies and the life quality of CC patients, the overall 5-year survival rate remains poor at 31% in China, owing to the delayed diagnosis, recurrence, and metastasis of tumor cells [5]. Tumor growth is fundamental for cancer development, so tumor growth suppression has always been a main therapeutic target for cancer treatment [1]. Hereby, identifying crucial molecular mechanisms implicated in growth and metastasis is of great importance for the exploration of therapeutic options optimizing the prognosis of CC patients.

Non-coding RNAs have attracted the researchers owing to the mediating roles in the biological behaviors of tumor cells [6]. Long non-coding RNAs (lncRNAs) and microRNAs (miRNAs) are two major classes of non-coding RNAs that are well recognized for their involvement in multiple processes through regulation on gene expression [7]. LncRNAs, defined as transcribed RNA over 200 molecules long, are commonly dysregulated in multiple cancers and play a variety of roles in tumorigenesis [8, 9], including in CC [10]. Several lncRNAs have been documented to regulate the growth and metastasis of CC [11, 12]. LncRNA differentiation antagonizing non-protein-coding RNA (DANCR) has recently been found to act as an oncogenic driver in several cancer type and correlated with tumor growth and metastasis [13]. Importantly, abnormal up-regulation of DANCR has been revealed to be linked with advanced tumor progression of CC [14]. But the molecular mechanisms of DANCR in CC remain largely unknown. The competing endogenous RNA (ceRNA) theory, which was initially raised by Salmena and his coworkers, proposes that non-coding and RNAs and protein-coding RNAs function as ceRNAs through competitively binding with miRNAs via shared miRNA recognition elements to regulate their expression [15]. DANCR has been documented to participate in several ceRNA networks and mediate tumorgenesis via sponging several mRNAs. For instance, DANCR has been suggested to induce the proliferation and metastasis of pancreatic cancer through mediating the miR-135a/NLRP37 axis [16]. Likewise, DNACR has been documented to sponge miR-135a to promote drug resistance of prostate cancer cells [17]. But the DANCR-associated ceRNA network in CC is yet unclear. Therefore, the current study was designed to validate the role of DANCR in CC progression and the molecular mechanisms involved.

## Methods

### Clinical sample collection

CC and paracancerous tissues (over 2 cm away from the CC tissues) were collected from 69 CC patients (46 males, 23 females, medium age 63.8 years and mean age 65.1 ± 7.2 years) who diagnosed and admitted in HwaMei Hospital, University of Chinese Academy of Sciences from June 2012 to July 2013. Followed-up studies on all patients were performed every 3 months for a total of 5 years. The patients were included if: 1) they were diagnosed as CC via the pathological examination; 2) they had never undergone radiotherapy or chemotherapy before surgery; 3) they had compete clinical information. The patients were excluded if: 1) they had chronic system diseases; 2) they had other malignant tumors. The study was ratified and supervised by the Clinical Ethical Committee of HwaMei Hospital, University of Chinese Academy of Sciences and in line with the *Declaration of Helsinki*. Signed informed consent was acquired from each eligible participant.

### Microarray analysis

Microarray analysis was conducted as a previous report [18]. In brief, total RNA from 5 pairs of CC and paracancerous tissues was extracted. Next, cDNA was synthesized using 0.5 μg total RNA via a GeneChip 3’In-vitro Transcription Express Kit (Thermo Fisher Scientific Inc., Waltham, MA, USA, 902789). Then the cDNA was fragmented and hybridized with human LncRNA Array V3.0 (Arraystar Inc., USA, AS-LNC-H-V4.0). After that, the chips were washed and scanned using a GeneChip™ Scanner 3000 7G system (Thermo Fisher, 000213).

### Reverse transcription quantitative polymerase chain reaction (RT-qPCR)

Total RNA was extracted from CC tissues and cells with RNAiso Plus (Takara Shuzo Co. Ltd., Otsu, Shiga, Japan) and Trizol LS Reagent (Takara), respectively. Then the qualified RNA was validated using formaldehyde gel electrophoresis and used for following experiments. Next, reverse transcription was conducted using a PrimeScript™ RT kit (Takara) [19] as per the manufacturer’s protocol, and real-time qPCR was conducted to quantify the mRNA expression using a SYBR Premix Ex Taq Kit (Takara). U6 and glyceraldehyde-3-phosphate dehydrogenase were served as internal references. The primer sequences are listed in Table 1.

**Table 1:** Primers used in RT-qPCR

Gene	Forward (5′-3′)	Reverse (5′-3′)
DANCR	CTGCATTCCTGAACCGTTATCT	GGGTGTAATCCACGTTTCTCAT
MDM2	GACCGAGTCTTGCTCTGTTACCC	GAGCGTGTCTTGCTCTCTTTCCC
GAPDH	CCCTGCTCTGGTTTGGTGAG	GAAGGCGTCTGAGGACTTAAA
MiR-518a	ACAGGCCGGGACAAGTGCAATA	GCTGTCAACGATACGCTACGTAACG
U6	AACGCTTCACGAATTTGCGT	CTCGCTTCGGCAGCACA
LINC005369	TCAGGATTCAGTTTCAGATCAG	CATTTCCAATAGTCAGCTAAGG
PCYT1B-AS1	GGTGAACTGAAATGTTAGCCCAG	AGGAGATTTGTTTGGCGTGC
LINC-PINT	GGTGAACTGAAATGTTAGCCCAG	GATTGGCTACCCAACTGTTG
MIR4438-2HG	CAGGGGCAGCAGCCACAAA	TAGGCGGTTGAATGAGAGG

### Cell culture transfection

Human CC cell lines HT29 (RRID: CVCL_0320), HCT116 (RRID: CVCL_0291), SW116 (RRID: CVCL_0544) and Caco-2 (RRID: CVCL_0025) and normal colon epithelial cell line FHC (RRID: CVCL_3688) were acquired from ATCC (Manassas, VA, USA). The cells were seeded into culture dishes at 1 × 10^5^ cells/cm^2^, and then filled with Roswell Park Memorial Institute-1640 (Gibco Company, Grand Island, NY, USA) supplemented with 10% fetal bovine serum (FBS) for 48 h of incubation at 37 °C with 5% CO_2_. The cells were detached with 0.025% trypsin (Gibco) and passaged when the cell confluence reached 80–90%.

Well-growing HT29 cells were collected and allocated into scramble group (cells were transfected with 100 pmol scramble small interfering (si) RNA), si-DANCR group (cells were transfected with 100 pmol DANCR-siRNA), si-DANCR + mock group (cells were co-transfected with mock and DANCR-siRNA, 100 pmol for each), si-DANCR + miR-518a-3p group (cells were co-transfected with miR-518a-3p mimic and DANCR-siRNA, 100 pmol for each), si-DANCR + empty vector (EV) group (cells were co-transfected with EV and DANCR-siRNA, 100 pmol for each) and si-DANCR + murine double minute 2 (MDM2) group (cells were co-transfected with MDM2 vector and DANCR-siRNA, 100 pmol for each). Likewise, well-growing SW116 cells were assigned into scramble, si-DANCR, si-DANCR + mock, si-DANCR + miR-518a-3p, si-DANCR + EV and si-DANCR + MDM2 groups after corresponding transfection as performed in HT29 cells. All transfection was performed as per the manufacturer’s instructions of a Lipofectamine™ 3000 kit (Invitrogen Inc., Carlsbad, CA, USA). The siRNA, miR-518a-3p inhibitor and the MDM2 EV are presented in Table 2.

**Table 2:** Sequence of siRNA/vector/inhibitor used in cell transfection

Gene	Sequence (5′-3′)
DANCR siRNA-1	AGCCAACTATCCCTTCAGT
DANCR siRNA-2	GAGCTAGAGCAGTGACAAT
MDM2	CCAGCTGGAGACAAGTCAGG
MiR-518a-3p inhibitor	ACTAGTTAT AACCCTAGGAATTTAGACAAC

### Western blot analysis

Total protein from cells was extracted using RIPA lysis buffer containing protease inhibitor. The protein content in supernatant was detected via bicinchoninic acid method. Next, an equal volume of protein (50 mg) was loaded on 10% sodium dodecyl sulfate-polyacrylamide gel electrophoresis (Bio-Rad, Hercules, CA, USA) and transferred onto polyvinylidene fluoride membranes (Millipore, Billerica, MA, USA). Then the membranes were incubated in tris-buffered saline tween with 5% skim milk at room temperature to block non-specific binding. After that, the membranes were incubated with primary antibodies at 4 °C overnight, and further with secondary antibody at room temperature for 1 h. Then the protein bands were visualized and imaged using the BioSpectrum system (Bio-Rad). The antibodies are shown in Table 3.

**Table 3:** Antibodies for western blot assay

Antibody	Item No.	Dilution ratio
E-cadherin	ab1416	1: 100
Vimentin	ab119139	1: 100
MDM2	ab38618	1: 5000
Snail	ab53519	1: 50
β-actin	ab179467	1: 5000
Smad 2	ab33875	1:2000
Smad 3	ab40854	1:2000
Secondary antibody	ab150117	1: 5000

Note: all antibodies were purchased from Abcam Inc., Cambridge, MA, USA

### 3-(4, 5-dimethylthiazol-2-yl)-2, 5-diphenyltetrazolium bromide (MTT) assay

Exponentially growing cells were trypsinized and diluted to 1 × 10^4^/mL single cell suspension. Then the suspension was loaded into 96-well plates at 2 μL per well (2 × 10^3^ cells), with plates filled with culture solution only set as control. The cells were incubated at 37 °C with 5% CO_2_ for 0–3 d. Each well was loaded with 20 μL MTT (5 mg/mL) solution at 0 h, 24 h, 48 h and 72 h, respectively, followed by 4 h of further incubation. Next, each well was loaded with 200 μL dimethyl sulfoxide and vibrated for 10 min to fully dissolved the crystals, and then the optical density (OD) value at 490 nm was measured.

### 5-ethynyl-2′-deoxyuridine (EdU) labeling assay

Well-growing passage 3 cells were collected and the DNA replication capacity of the cells was detected using a Cell-Light EdU labeling kit (RiboBio Co., Ltd., Guangzhou, Guangdong, China) as per the instructions [20]. Five random fields were photographed under a fluorescence microscope (Olympus Optical Co., Ltd., Tokyo, Japan, FSX100), in which the blue fluorescence indicates total cells while the red refers the replicating cells with EdU labeling. The EdU-positive cell rate was measured.

### Detection of invasion and migration of HT29 and SW116 cells

Transwell assays were applied for cell invasion detection. Firstly, the apical chambers were pre-coated with Martrigel (BD Biosciences, Franklin Lakes, NJ, USA) under sterile condition for 30 min, and then each chamber was filled with 30 μL RPMI-1640 and placed in a CO_2_ incubator. HT29 and SW116 cells were detached, centrifuged, resuspended in serum-free medium, and diluted to cell suspension at 5 × 10^5^cells/mL. Next, each basolateral chamber was filled with 500 μL 10% FBS-supplemented RPMI-1640, while each apical chamber was loaded with 200 μL cell suspension. Then the chambers were incubated in a 37 °C incubator with 5% CO_2_ for 48 h. Next, the chambers were taken out with the medium washed away by PBS, and the invaded cells were stained by crystal violet for 10 min and then had the superficial crystal violet rinsed away. The non-invaded cells in the apical chambers were wiped away using cotton swabs, and the invaded cells were photographed under the microscope and the cell number was calculated.

Migration of HT29 and SW116 cells was performed via Transwell assay as well and all the procedures were conducted as above stated but without pre-coating Matrigel in the apical chambers. The chambers were taken out for staining after 24 h of incubation.

### Subcellular localization of lncRNA DANCR

Subcellular localization of DANCR was analyzed at Lncatlas (http://lncatlas.crg.eu/) and further identified with fluorescence in situ hybridization (FISH) method using a Ribo™ lncRNA FISH Probe Mix (Green) (RiboBio) according to the manufacture’s protocol. After that, the nuclear and cytoplasmic RNA was separated following the instructions of a PARIS™ Kit (Life Technologies, Inc., Gaithersburg, MD, USA) to further confirm the distribution of DANCR in cells.

### Dual-luciferase reporter gene assay

The binding sites between miR-518a-3p and DANCR and between miR-518a-3p and the 3′-untranslated region (3′ UTR) of MDM2 were predicted on StarBase (http://starbase.sysu.edu.cn/) [21]. Then the pMIR-REPORT™-based DANCR-wild type (WT) and DANCR-mutant type (MUT) plasmids as well as pMIR-REPORT™-based MDM2-WT and MDM2-MUT plasmids were synthetized by Sangon Biotech (Shanghai) Co., Ltd. (Shanghai, China) [22]. Well-designed WT and MUT plasmids were co-transfected with either miR-518a-3p mimic or miR NC into 293 T cells (RRID: CVCL_0063; ATCC) using the Lipofectamine™ 3000 kit. Cells were lysed 24 h later, and the relative luciferase activity was measured using a Dual-Luciferase Reporter Assay System (Promega Corporation, WI, USA).

### Biotinylated RNA pull-down assays

Cell lysates were treated with RNase-free DNase I (Sigma-Aldrich Chemical Company, St Louis, MO, USA) and incubated with a mixture of biotinylated RNA fragments of miR-518a-3p (1 μg) and streptavidin-coated magnetic beads (Sigma-Aldrich) at 4 °C for 3 h. The RNA was extracted from the captured RNA-RNA complexes for western blot analysis.

### Hoechst 33258 staining

Well-growing cells were fixed in 4% paraformaldehyde for 20 min and then stained with Hoechst 33258 (10 μg/mL) solution for 5 min. Then the cells were observed under the fluorescence microscope with 5 fields randomly selected.

### Flow cytometry

Well growing HT29 and SW116 cells in each group were stained with Annexin V- fluorescein isothiocyanate (FITC) and propidium iodide (PI) as per the instructions of an Annexin V-FITC/PI apoptosis detection kit (Keygen Biotech CO., Ltd., Nanjing, Jiangsu, China). The cell apoptosis rate was analyzed using a flow cytometer (FACSCanto II, BD Biosciences, San Jose, CA, USA). Three independent experiments were performed.

### Xenograft tumor models

Twenty-four specific-pathogen-free grade nude mice (BALB/c, 4–6 weeks old, 18–22 g, Laboratory Animal Center, Chinese Academy of Sciences, Shanghai, China) were numbered by weight and allocated into 4 groups, 6 in each. Thereafter, 4 × 10^6^ HT29 and SW116 cells from the scramble and si-DANCR groups were dispersed with 2 mL saline and subcutaneously injected into the four groups of mice, correspondingly. The volume of tumor in mice was measured every 7 d as the formula: m_1_^2^ × m_2_ × 0.5236 [23], in which m_1_ refers to the minor axis while m_2_ refers to the major axis. The mice were euthanized via overdose of pentobarbital on the 35th d, and the tumors were weighed and collected for histology experiments. Animal experiments were performed in compliance with the recommendations in the Guide for the Care and Use of Laboratory Animals of the National Institutes of Health. The protocol was approved by the Animal Ethics Committee of HwaMei Hospital, University of Chinese Academy of Sciences. Great efforts were made to minimize the number and suffering of animals.

### Immunohistochemical staining

Tumor tissues from each group of mice were embedded in paraffin, dewaxed, and dehydrated. Each tissue was cut into 5 sections. The sections were washed with PBS for 3 times, added with 3 drops of H_2_O_2,_ and then allowed to stand at room temperature for 15 min. Next, the sections were incubated with 50 μL rabbit anti-human KI67 (1: 500, ab15580, Abcam Inc., Cambridge, MA, USA) at 4 °C overnight. Following 3 PBS washes, the sections were cultured with secondary antibody rabbit-anti mouse immunoglobulin G (IgG, ab150117, Abcam) at 37 °C for 20 min, washed with PBS for 3 times, and further incubated with 50 μL HRP-labeled streptavidin at 37 °C for 20 min. Following PBS washes, the sections were visualized using diaminobenzidine, washed with distilled water, counterstained with hematoxylin for 30 s, dehydrated, and sealed with neutral balsam. The sections were then observed under the microscope, under which the KI67-positive cells presented brown or yellow particles in the nuclei. To each section, 5 nonoverlapping fields were selected, and the number of KI67-positive cells was calculated.

### Tumor metastasis in nude mice

Twenty-four nude mice (the same batch as the above ones) were allocated into 4 groups, 6 mice in each, and each mouse was injected with 4 × 10^6^ HT29 or SW116 cells from the scramble and si-DANCR groups through the caudal vein. Mice were sacrificed on the 45^th^ d with the lung and liver tissues extracted for hematoxylin and eosin (HE) staining, which was performed as previously reported [24].

### Statistical analysis

SPSS 21.0 (IBM Corp. Armonk, NY, USA) was used for data analysis. Data were in normal distribution according to Kolmogorov-*Smir*nov method and described as mean ± standard deviation (mean ± SD). Differences between each group pair were measured using the *t*-test while differences among multiple groups were compared with one-way analysis of variance (ANOVA) or two-way ANOVA. Tukey’s multiple comparisons test was used for the pairwise comparison after ANOVA. Survival curve was drawn via the Kalpan-Meier method and analyzed using log rank test. Enumeration data were compared by Fisher’s exact test. *p* was obtained by two-tailed test and *p* < 0.05 was regarded to show a statistically significant difference.

## Results

### DANCR is highly expressed in CC patients and is correlated with poor prognosis

**Fig. 1:**
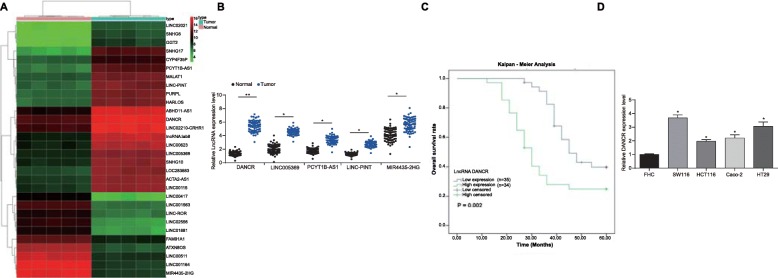
DANCR is highly expressed in CC patients and is correlated with poor prognosis. Microarray analysis was performed between normal tissues and tumor tissues by Arraystar Human LncRNA microarray V2.0 (Agilent_033010 Probe Name version). **a**, heatmap for 30 differentially expressed lncRNAs; **b**, 5 mostly changed lncRNAs between normal tissues and tumor tissues detected assessed using RT-qPCR; **c**, Kaplan-Meier survival analysis of CC patients with high (*n* = 35) or low DANCR expression (*n* = 34); **d**, DANCR expression in normal colon epithelial cell line FHC and CC cell lines measured using RT-qPCR. Data are expressed as mean ± SD; in panel B, data were analyzed using the paired *t*-test, data in panel D were analyzed using one-way ANOVA and Tukey’s multiple comparison test; *, *p* < 0.05

Five pairs of CC and paracancerous tissues were collected for microarray analysis. We found a total of 221 differentially expressed lncRNAs, among which 116 were up-regulated while 95 were down-regulated in CC tissues, with the top 30 changed lncRNAs presented in the Heatmap (all *p* < 0.05) (Fig. 1a). To further validate the results of microarray analysis, 5 mostly changed lncRNAs in 69 pairs of CC and paracancerous tissues were assessed using RT-qPCR, which showed same trends as the microarray analysis (all *p* < 0.05, Fig. 1b). LncRNA DANCR, which held the greatest changing degree, was selected as our study subject. Next, the DANCR expression in all 69 CC patients was evaluated, and the patients were further assigned into high-DANCR expression group and low-DANCR group based on the medium level (5.49). According to the follow-up studies on the CC patients and Kalpan-Meier survival analysis, it was found that CC patients with higher DANCR expression had worse prognosis and less survival time (*p* < 0.05) (Fig. 1c). We further explored DANCR expression in normal colon epithelial cell line FHC and CC cell lines SW116, HCT116, Caco-2 and HT-29 using RT-qPCR, which suggested that DANCR expression was notably higher in CC cell lines than that in FHC cells (all *p* < 0.05, Fig. 1d).

### Silencing of DANCR reduces the malignant behaviors of CC cells

**Fig. 2:**
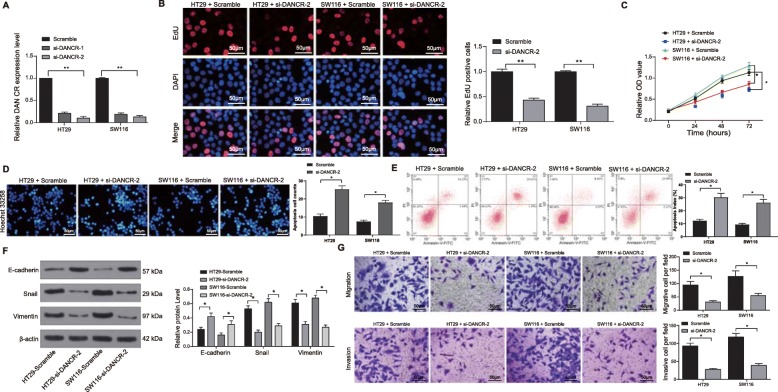
Silencing of DANCR reduces the malignant behaviors of CC cells. si-DANCR plasmids were transfected into HT29 and SW116 cells with scramble siRNA as NC. **a**, DANCR expression following si-DANCR-1 and si-DANCR-2 plasmid transfection detected using RT-qPCR; **b**, proliferation of HT29 and SW116 cells measured via EdU assay; **c**, viabilities of HT29 and SW116 cells detected using MTT assay; **d**-**e**, apoptosis of HT29 and SW116 cells evaluated using Hoechst 33258 staining (D) and flow cytometry (E); **f**, protein levels of EMT markers Snail, Vimentin and E-cadherin in cells determined by western blot analysis (See original images in Supplementary Figure S1); **g**, invasion and migration abilities of HT29 and SW116 cells assessed via Transwell assays. Data are expressed as mean ± SD; in panels A, B, D, E and F, data were analyzed using one-way ANOVA, while data in panels C and F were analyzed via two-way ANOVA, and Tukey’s multiple comparison test was applied for the post hoc test; *, *p* < 0.05

To further identify the roles of DANCR in CC cell behaviors, DANCR expression in cells was interfered with siRNA. Well-constructed si-DANCR-1 and si-DANCR-2 plasmids were transfected into HT29 and SW116 cells, after which we found DANCR expression was down-regulated, and the si-DANCR-2 plasmid showed a higher interfering efficacy (all *p* < 0.05) (Fig. 2a).

Next, EdU and MTT assays were applied to measure cell proliferation. The results told that DANCR inhibition reduced proliferation of HT29 and SW116 cells (all *p* < 0.05, Fig. 2b-c). Meanwhile, Hoechst 33258 staining results suggested that the apoptosis of HT29 and SW116 cells was improved following DANCR inhibition (all *p* < 0.05) (Fig. 2d). Likewise, the flow cytometry presented an increased ratio in apoptotic cells after DANCR silencing (Fig. 2e).

The levels of epithelial mesenchymal transition (EMT) marker proteins in HT29 and SW116 cells were measured via western blot analysis. Silencing of DANCR led to a decrease in protein levels of Vimentin and Snail but an increase in protein level of E-cadherin (all *p* < 0.05) (Fig. 2f). Moreover, the Transwell assays suggested that the invasion and migration abilities of HT29 and SW116 cells were decreased following DANCR inhibition (all *p* < 0.05, Fig. 2g).

### DANCR regulates MDM2 expression via interacting with miR-518a-3p

**Fig. 3:**
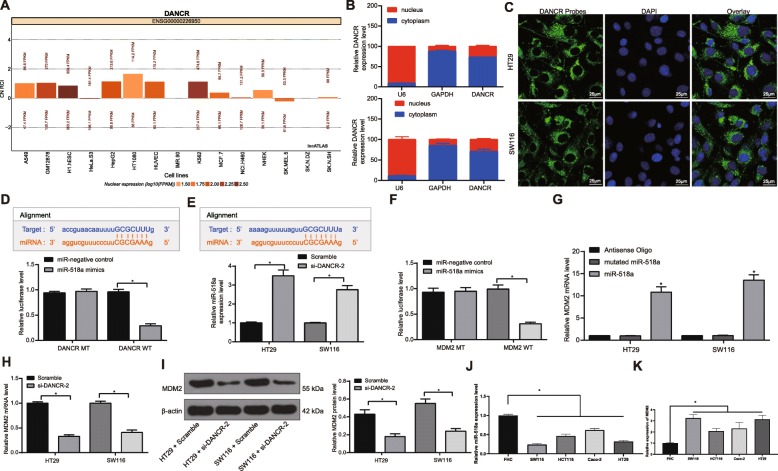
DANCR regulates MDM2 expression via interacting with miR-518a-3p. **a**, subcellular localization of DANCR predicted via the Lncatlas database; **b**, nuclear and cytoplasmic DANCR expression in HT29 and SW116 cells determined by RT-qPCR; **c**, FISH experiments with probes targeting DANCR were performed to validate the subcellular localization of DANCR in HT29 and SW116 cells, the cytoplasm was stained with probes targeting DANCR (green stain), and the nuclei were stained with DAPI (blue stain); **d**, binding relationship between DANCR and miR-518a-3p were predicted on StarBase, and luciferase reporter plasmids containing DANCR-WT or DANCR-MUT were co-transfected into H293T cells with miR-NC plasmid as NC; **e**, miR-518 expression in HT29 and SW116 cells following DANCR inhibition detected using RT-qPCR; **f**, binding relationship between miR-518a-3p and MDM2 were predicted on StarBase luciferase reporter plasmids containing MDM2-WT or MDM2-MUT were co-transfected into H293T cells with miR-NC plasmid as NC; **g**, enrichment of miR-518a-3p on the MDM2 mRNA detected by RNA pull down-qPCR assay, relative to antisense-oligos; **h**-**i**, relative mRNA expression (H) and protein level (I) of MDM2 following DANCR inhibition determined by qRT-PCR and western blot analysis (See original images in Supplementary Figure S2), respectively; **j**-**k**, expression of miR-518a-3p and MDM2 in HT29 and SW116 cells and in FHC cells determined by RT-qPCR. Data are expressed as mean ± SD; in panels D, E, G, H, J and K, data were analyzed using one-way ANOVA, while data in panels B, I and F were analyzed via two-way ANOVA, and Tukey’s multiple comparison test was applied for the post hoc test; *, *p* < 0.05, **, *p* < 0.01

The Lncatlas website predicted that DANCR is mainly sub-localized in cytoplasmic matrix (Fig. 3a). Then the FISH and nuclear/cytoplasmic RNA-separation experiments showed that DANCR was mainly localized in cytoplasm in HT29 and SW116 cells (Fig. 3b-c), indicating that DANCR might exert functions through the ceRNA network.

miR-518a-3p was selected as a DANCR target according to the predictions on StarBase, The binding relationship between DANCR and miR-518a-3p was validated through the dual luciferase reporter gene assay (all *p* < 0.05) (Fig. 3d). Then we measured miR-518 expression in CC cells, and the results showed that miR-518a-3p expression was up-regulated following DANCR inhibition (*p* < 0.05) (Fig. 3e). Thereafter, we further explored the target genes of miR-518a-3p on StarBase. We turned the focus on MDM2, since it has been documented that miR-518 could bind to MDM2 and then inhibit proliferation, metastasis and drug-resistance of gastric cancer cells [25]. Therefore, dual luciferase gene reporter and RNA-pull down assays were performed, and it was found that miR-518 could directly bind to the 3′-UTR of the MDM2 mRNA (all *p* < 0.05) (Fig. 3f-g). Moreover, RT-qPCR and western blot analysis results suggested that silencing of DANCR decreased the mRNA and protein levels of MDM2 in HT29 and SW116 cells (all *p* < 0.05) (Fig. 3h-i). In addition, expression of miR-518a-3p and MDM2 mRNA in CC cells and FHC cells was determined. The results told that miR-518a-3p expression was decreased while MDM2 expression was increased in CC cell lines versus in normal FHC cells (Fig. 3j-k).

### Silencing of miR-518a-3p partially reverses the inhibition on CC cell behaviors induced by DANCR down-regulation

**Fig. 4:**
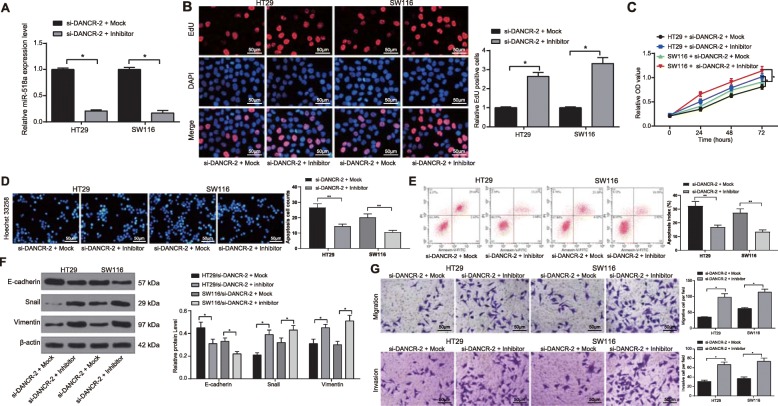
Silencing of miR-518a-3p partially reverses the inhibition on CC cell behaviors induced by DANCR down-regulation. miR-518a-3p inhibitor and its mock plasmid were transfected into si-DANCR-treated HT29 and SW116 cells. **a,** miR-518a-3p expression in cells measured using RT-qPCR; **b**, proliferation of HT29 and SW116 cells measured via EdU assay; **c**, viabilities of HT29 and SW116 cells detected using MTT assay; **d**-**e**, apoptosis of HT29 and SW116 cells evaluated using Hoechst 33258 staining (D) and flow cytometry; **f**, protein levels of EMT markers Snail, Vimentin and E-cadherin in cells determined by western blot analysis (See original images in Supplementary Figure S3); **g**, invasion and migration abilities of HT29 and SW116 cells assessed via Transwell assays. Data are expressed as mean ± SD; in panels A, B, D, E and G, data were analyzed using one-way ANOVA, while data in panels C and F were analyzed via two-way ANOVA, and Tukey’s multiple comparison test was applied for the post hoc test; *, *p* < 0.05

To confirm the roles of miR-518a-3p in CC cell behaviors, we further transfected CC cells with miR-518a-3p inhibitor after DANCR inhibition. Then it was found that down-regulation of miR-518a-3p reversed the inhibitory effects of DANCR silencing on CC cells. The proliferation, viability, invasion and migration of cells were elevated, while the apoptosis of cells was decreased following further miR-518a-3p inhibition (all *p* < 0.05) (Fig. 4a-g).

### Overexpression of MDM2 partially reverses the inhibition on CC cell behaviors induced by DANCR silencing

**Fig. 5:**
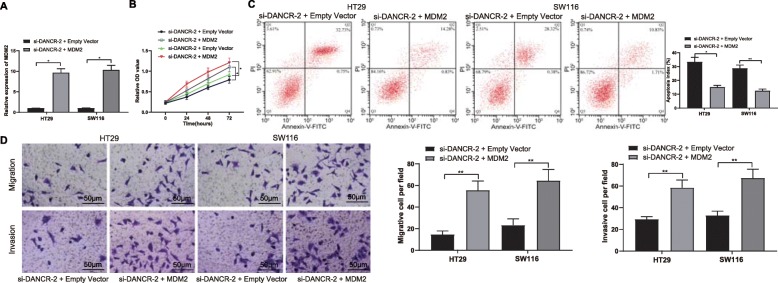
Overexpression of MDM2 partially reverses the inhibition on CC cell behaviors induced by DANCR silencing. MDM2 overexpressing vector and the corresponding EV were transfected into HT29 and SW116 cells with stably silenced DANCR. **a**, MDM2 expression in cells determined using RT-qPCR; **b**, proliferation activity of HT29 and SW116 cells measured using MTT assay; **c**, the ratio of apoptotic cells determined by flow cytometry; **d**, migration and invasion abilities of cells evaluated using Transwell assays. Data are expressed as mean ± SD; in panels A, C and D, data were analyzed by one-way ANOVA, while data in panel B by two-way ANOVA, and Tukey’s multiple comparison test was applied for the post hoc test; *, *p* < 0.05

To clarify the roles of MDM2 in CC progression, artificial overexpression of MDM2 was further administrated by transfecting MDM2 overexpressing vector in CC cells with stable silenced DANCR (Fig. 5a). Then, it was found that overexpression of MDM2 led to a significant increase in cell invasion and migration but a decrease in number of apoptotic cells (Fig. 5b-d).

### Silencing of DANCR affects activation of the Smad2/3 and p53 signaling pathways

**Fig. 6:**
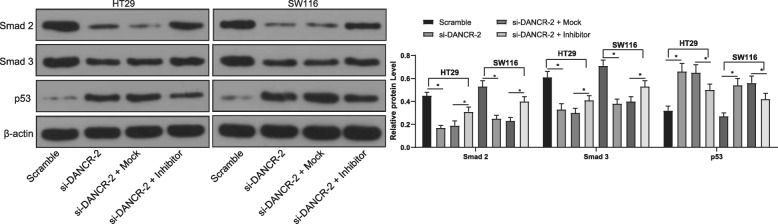
Silencing of DANCR inactivates the Smad2/3 signaling pathway. Protein levels of Smad2, Smad3 and p53 in HT29 and SW116 cells were measured using western blot analysis (See original images in Supplementary Figure S4). Data are expressed as mean ± SD; were analyzed via two-way ANOVA, and Tukey’s multiple comparison test was applied for the post hoc test; *, *p* < 0.05

MDM2 has been documented to activate the Smad2 /3 signaling pathway to promote the EMT of lung adenocarcinoma cells [26]. The Smad2/3 signaling pathway is activated in several human malignancies and has been documented to promote the renewal of CC stem cells [27]. In addition, MDM2 is a main negative regulator of p53, which is responsible for growth arrest and apoptosis [28]. Here, we proposed that the activation of Smad2/3 and p53 signaling pathway is involved in the MDM2-mediated events in CC. Then, we measured the protein levels of Smad2, Smad3 and p53 in HT29 and SW116 cells, with the findings that DANCR inhibition led to obviously reduced protein levels of Smad2 and Smad3 while increased level of p53. But the further miR-518a-3p inhibition reversed the above changes (all *p* < 0.05) (Fig. 6).

### Silencing of DANCR in CC cells inhibits tumor formation and metastasis in vivo

**Fig. 7:**
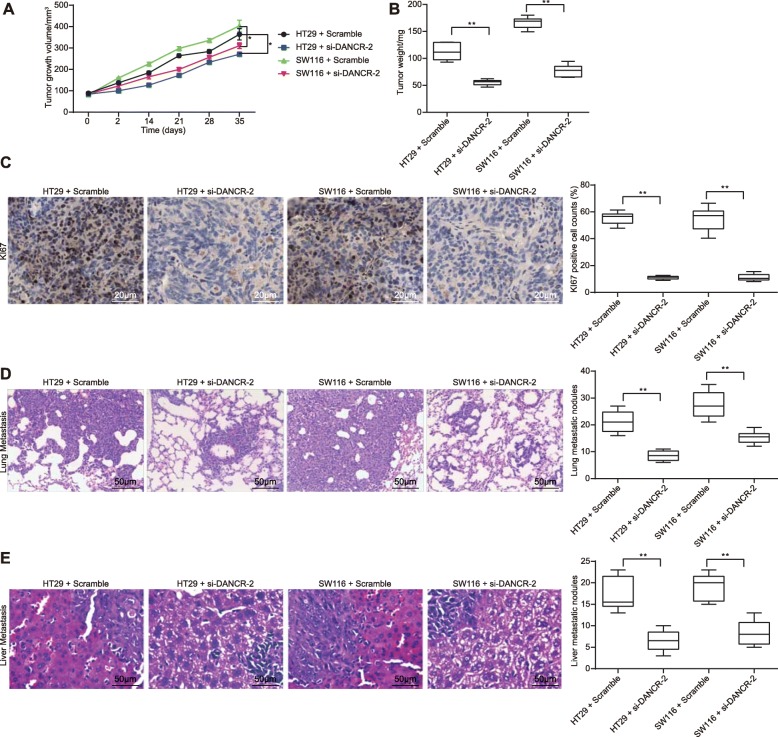
Silencing of DANCR in CC cells inhibits tumor formation and metastasis in vivo. HT29 and SW116 cells transfected with si-DANCR or scramble siRNA were subcutaneously inoculated into BALB/c nude mice at a dose of 4 × 10^6^ per mouse (*n* = 6 in each group). Tumor growth was measured continuously every 7 days. On the 35th d after implantation, the mice were euthanized via overdose of pentobarbital. **a**-**b** measurement of tumor size (A) and tumor weight (B); **c**, tumor sections were obtained and stained with and anti-KI67 antibodies, and the KI67-positive cells were evaluated using immunohistochemistry; **d**-**e**, another batch of mice (4 groups, each group *n* = 6) were injected with HT29 and SW116 cells through the tail vein, and the number of metastatic nodules in lung (D) and liver (E) was determined on the 45th d via HE staining. In panels B, C, D and E, data were analyzed using one-way ANOVA while data in panel A were analyzed using two-way ANOVA, and Tukey’s multiple comparison test was used for the post hoc test after ANOVA analysis, * *p* < 0.05

Following the findings from experiments in vitro, we further figured out the roles of DANCR silencing in nude mice. Cells transfected with si-DANCR or scramble siRNA were implanted into nude mice. In terms of tumor formation, our study found that silencing of DANCR inhibited the growth rate and the KI67-positive rate of the xenograft tumors in vivo (all *p* < 0.05) (Fig. 7a-c). In terms of tumor metastasis, it was shown that the formation of metastatic nodules in liver and lung were reduced when DANCR was down-regulated (all *p* < 0.05) (Fig. 7d-e).

## Discussion

CC treatment remains to be a huge challenge since most patients subjected to surgical resection hold quite high recurrence rate in a short period of nearly 2 years or moderately longer [29]. LncRNAs can function as ceRNAs to sponge miRNAs and stop these miRNAs from binding to the target mRNAs, thus mediating target genes posttranscriptionally [6]. Here we investigated the role of DANCR in CC progression, with the conclusion that silencing of DANCR could inhibit the growth and metastasis of CC cells through mediating miR-518a-3p and MDM2 expression and the further inactivation of the Smad2/3 signaling pathway.

The initial finding of our study was that DANCR was highly expressed in CC tissues and cells and it was correlated with poor prognosis of CC patients. Down-regulation of DANCR led to reduced proliferation, viability, invasion, migration, and resistance to death of CC cells, as well as reduced tumor formation and metastasis in vivo. High expression of DANCR has been recently found in human cancers [30] and DANCR acts as a tumor promoter in multiple malignancies such as ovarian cancer [31], gastric cancer [32], breast cancer [33] and so many like this. It has been documented that DANCR could promote the proliferation and invasion abilities of cancers [34, 35]. Importantly, the same trends have been found in CC, with DANCR up-regulation in CC tissues and resulted in poor outcome of CC patients [14]. Likewise, quite similar with our findings, it has been documented that silencing of DANCR promoted CC cell apoptosis while inhibited tumor growth [36]. Besides, DANCR silencing led to decreased protein levels of Vimentin and Snail but promoted level of E-cadherin in our study. Snail is well-known for the function in inducing EMT, during which the E-cadherin transcription is repressed during tumor progression, and the loss of E-cadherin in tumors is considered to lead poor clinical outcome [37]. Besides, Vimentin is an important contributor for EMT via regulating its linked genes [38]. These results further identified that down-regulation of DANCR reduced CC metastasis from the molecule perspective.

In light with the emerging evidence that lncRNAs might exert functions through the ceRNA networks, we explored the possible miRNAs mediated by DANCR in CC and found that miR-518a-3p could bind to DANCR through the online predictions and luciferase assay. DANCR silencing led to elevated miR-518a-3p expression, while down-regulation of miR-518a-3p promoted the malignant behaviors of CC cells. miR-518a-3p-5p has been suggested to target chemokine receptor CCR6 expression in CC cell lines and then to inhibit CC progression and invasion [39]. Similarly, miR-518p has been found to be lowly expressed in CC tissues and cells, and its up-regulation reduced cell proliferation and induced cell apoptosis [40]. The findings above triggered us to further confirm the gene holding accountability of the above events, and we found miR-518a-3p directly bound to the 3′-UTR of MDM2 mRNA, which was quite in coincidence with a previous report [25]. DANCR interacted with miR-518a-3p, and the DANCR inhibition led to reduced MDM2 expression in CC. MDM2, and its human homolog HDM2, are key negative mediators for p53 tumor suppressor protein and aberrantly highly expressed in several cancer types [41]. MDM2 inhibition was shown to induce growth arrest and DNA breakage in colon tumor in mouse and human CC cells [42]. The fact that overexpression of MDM2 partially reversed the inhibition on CC cell malignant behaviors by DANCR silencing further evidenced the involvement of MDM2 in the DANCR-mediated events. Moreover, our study found that silencing of DANCR reduced the protein levels of Smad2/3, which might be regulated by MDM2, since MDM2 has been documented to promote Smad2/3 activation in lung adenocarcinoma [26]. Smad2/3 are the key transducers of the termed transforming growth factor-β (TGF-β) signaling pathway, whose activation promotes tumor growth including CC [43]. Smad2/3 activation has been suggested to be closely linked with EMT of cancer cells [44, 45], while inhibited nuclear translocation of Smad2/3 has been suggested to participate in cancer cell apoptosis [46]. On the other hand, MDM2 is a main negative regulator of p53, which is a well-known tumor suppressor [28, 47]. Down-regulation of MDM2 and the destruction of MDM2-p53 interaction holds potential in cancer therapy [48]. To conclude, it can be inferred that activation of the Smad2/3 signaling and the p53 inhibition might be responsible for the DANCR/miR-518a-3p/MDM2-mediated CC progression.

## Conclusions

**Fig. 8:**
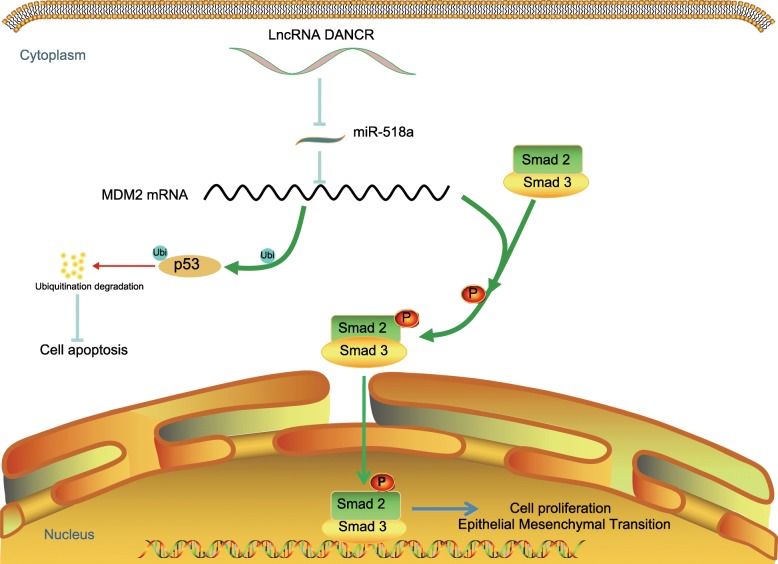
Diagram for the molecular mechanism. In CC cells, lncRNA DANCR elevates MDM2 expression through the crosstalk with miR-518a-3p. MDM2 further promotes the nuclear translocation of Smad2/3 and inhibits p53 expression, leading to growth and metastasis of CC cells

Taken together, the study provided evidence that silencing of DANCR might inhibit the growth and metastasis of CC cells through the DANCR/miR-518a-3p/MDM2 ceRNA network and the following Smad2/3 signaling inactivation (Fig. 8). The study yields novel insights into the lncRNA/miRNA/mRNA network in the progression of CC. These findings may offer new ideas for CC prevention and treatment. Also, we hope more studies in the near future would be performed to validate our findings and, to develop more therapeutic options for CC treatment.

## Supplementary information

**Additional file 1: Figure S1.** Original gels and blots of E-cadherin, Snail, Vimentin and β-actin (Corresponding to Fig. 2f in the manuscript). **Figure S2.** Original gels and blots of MDM2 and β-actin (Corresponding to Fig. 3i in the manuscript). **Figure S3.** Original gels and blots of E-cadherin, Snail, Vimentin and β-actin (Corresponding to Fig. 4f in manuscript). **Figure S4.** Original gels and blots of Smad2, Smad3, p53and β-actin of H29 and SW116 cells (Corresponding to Fig. 6 in manuscript).

## Data Availability

All the data generated or analyzed during this study are included in this published article.

## Abbreviations

ANOVA: Analysis of variance
CC: Colon cancer
CRC: Colorectal cancer
DANCR: Differentiation antagonizing non-protein-coding RNA
EdU: 5-Ethynyl-2′-deoxyuridine
ceRNA: Competing endogenous RNA
FBS: Fetal bovine serum
FISH: Fluorescence in situ hybridization
HE staining: Hematoxylin and eosin staining
IgG: Immunoglobulin G
lncRNA: Long non-coding RNA
MDM2: Murine double minute 2
miRNA: Microrna
MUT: Mutant type
MTT: 3-(4, 5-Dimethylthiazol-2-yl)-2, 5-diphenyltetrazolium bromide
PBS: Phosphate buffer saline
RT-qPCR: Reverse transcription quantitative polymerase chain reaction
SD: Standard deviation
siRNA: Small interfering RNA
WT: Wild type
3’UTR: 3′-Untranslated region
