# Supplementary material for: Roles of DANCR/microRNA-518a-3p/MDMA ceRNA network in the growth and malignant behaviors of colon cancer cells
Source: BMC Cancer. 2020 May 18;20:434. doi: 10.1186/s12885-020-06856-8 (PMC7236548; doi:10.1186/s12885-020-06856-8)

Supplementary Fig. S1. Original gels and blots of E-cadherin, Snail, Vimentin and β-actin (Corresponding to Fig. 2F in the manuscript)


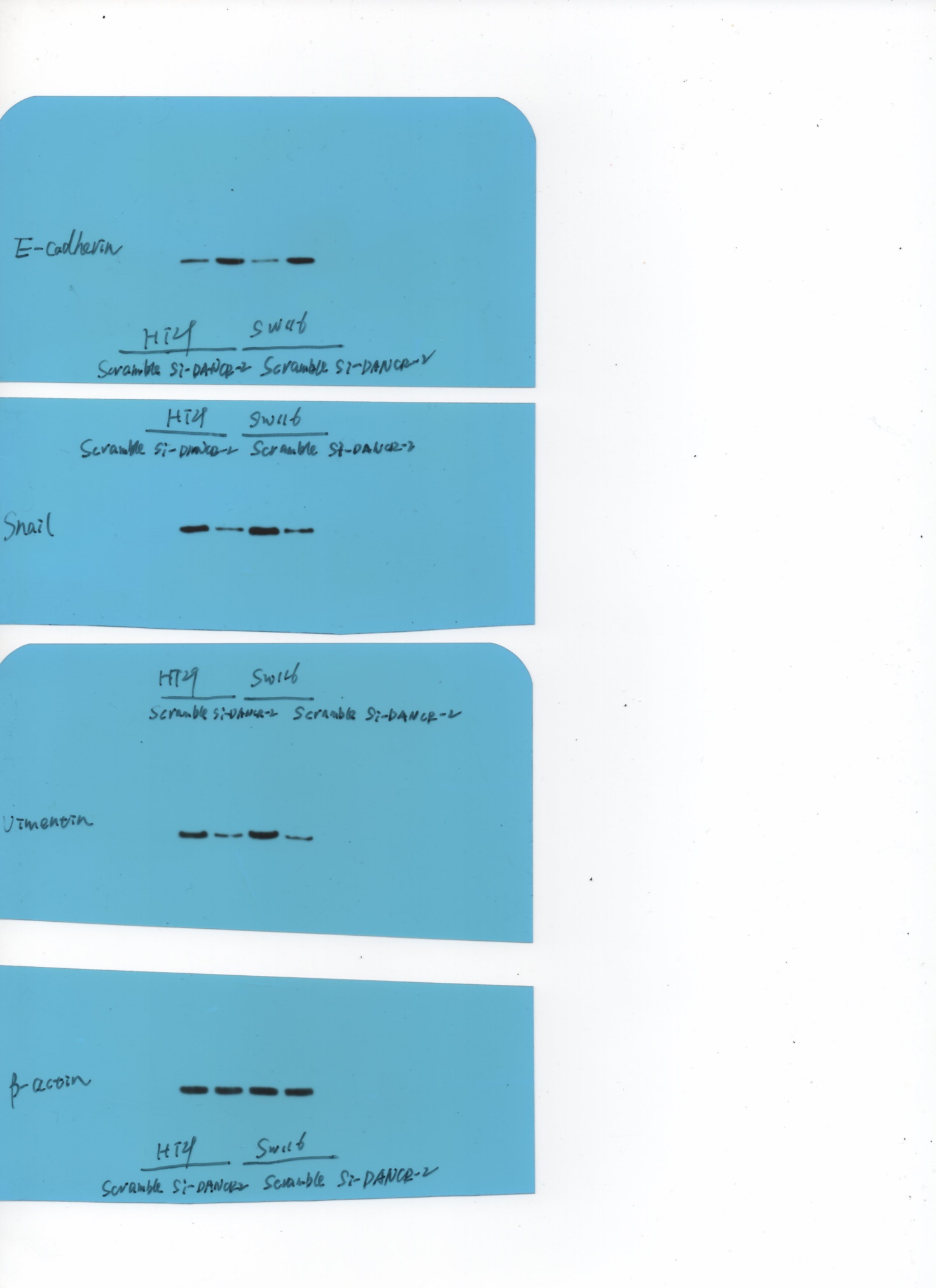


Supplementary Fig. S2. Original gels and blots of MDM2 and β-actin (Corresponding to Fig. 3I in the manuscript)


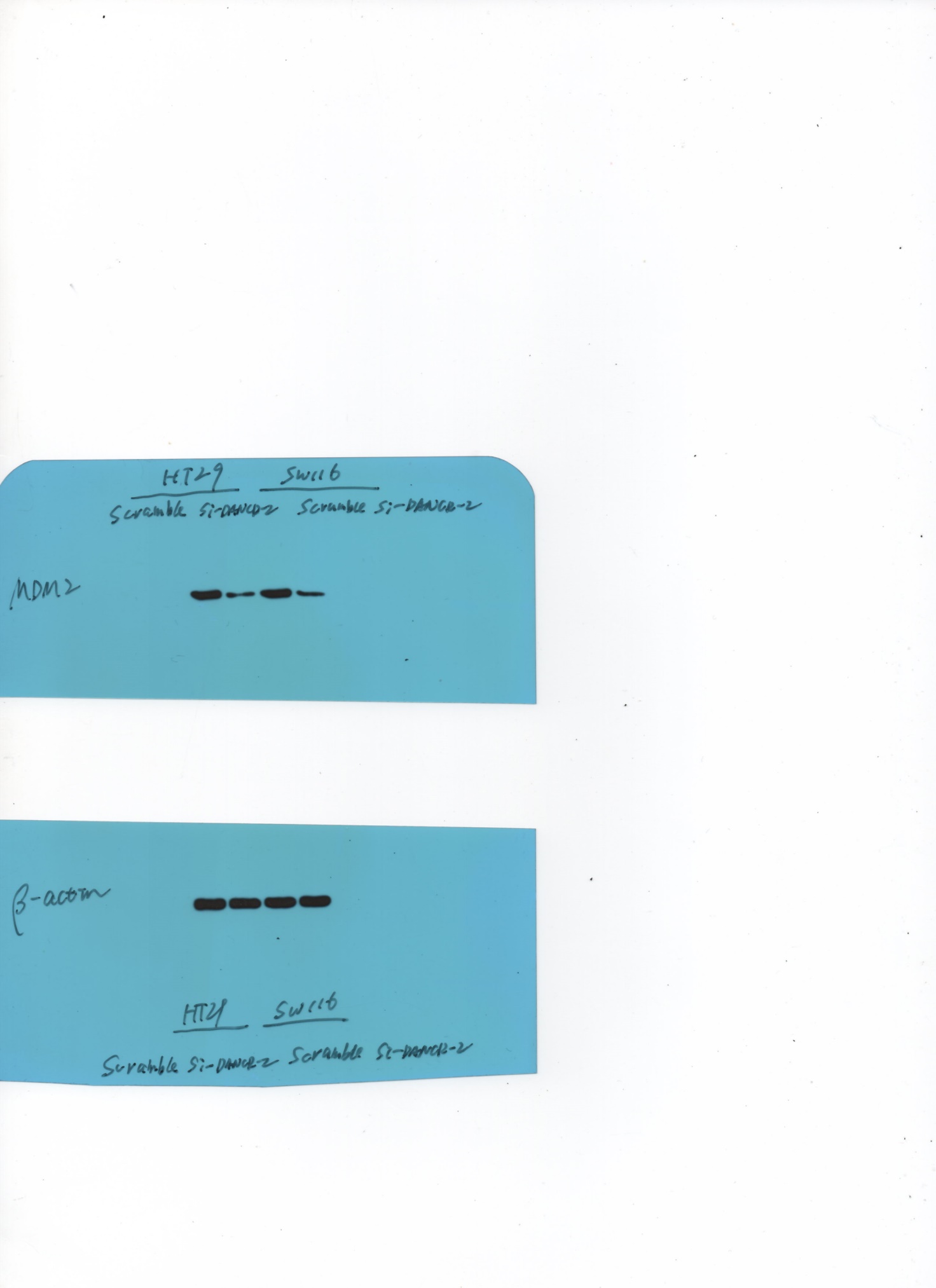


Supplementary Fig. S3. Original gels and blots of E-cadherin, Snail, Vimentin and β-actin (Corresponding to Figure 4F in manuscript)


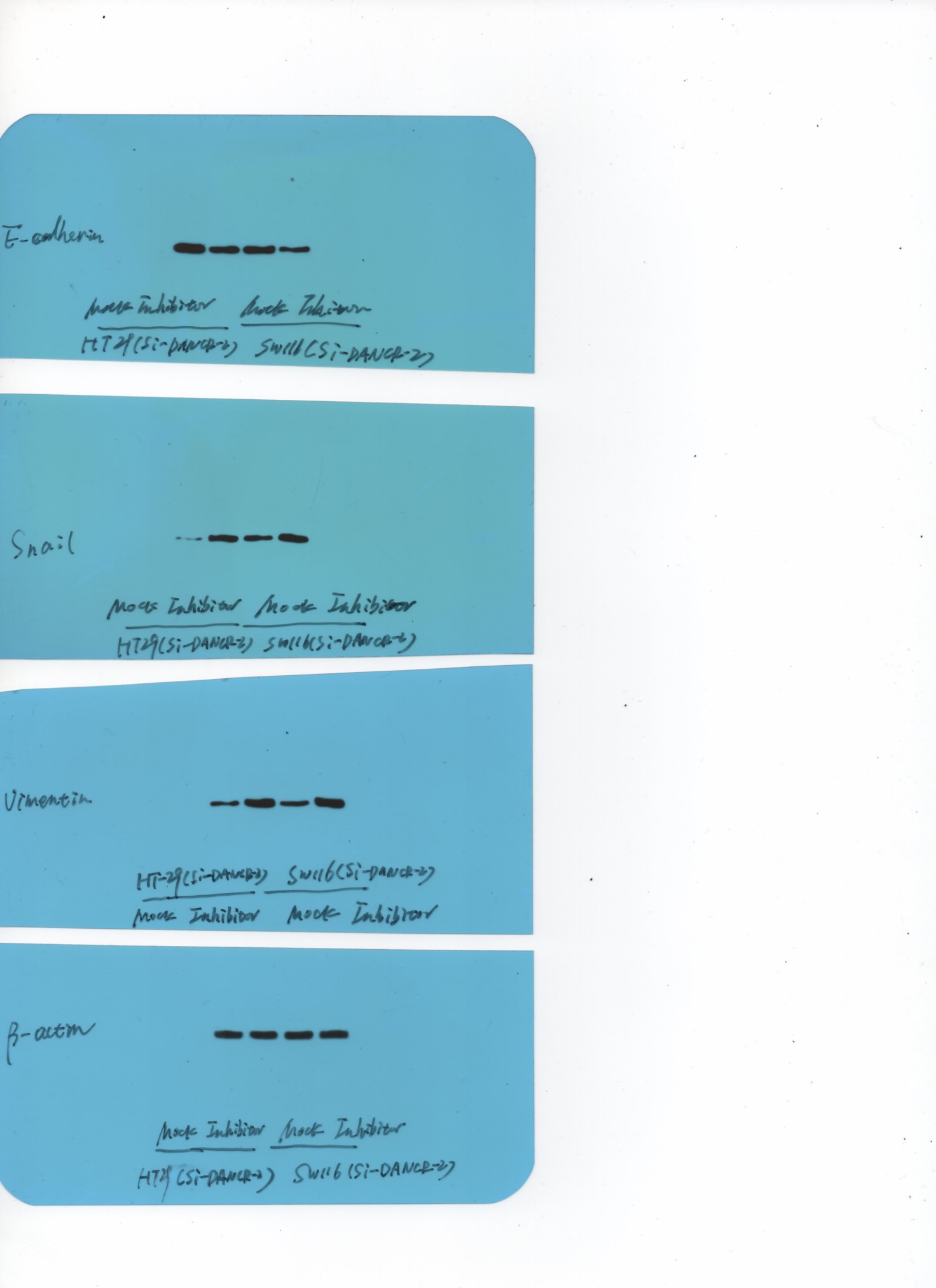


Supplementary Fig. S4. Original gels and blots of Smad2, Smad3, p53and β-actin of H29 and SW116 cells (Corresponding to Figure 6 in manuscript)


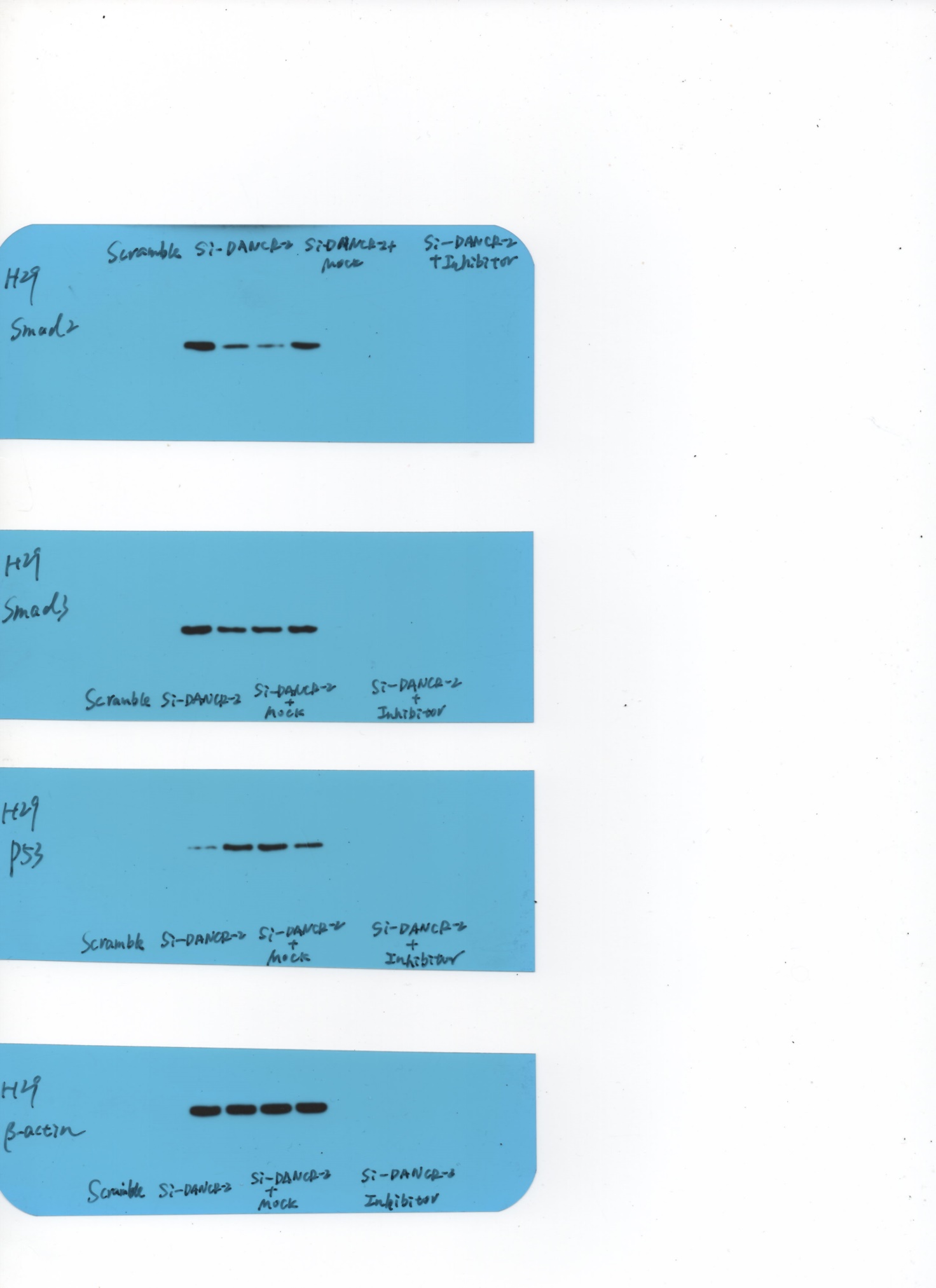


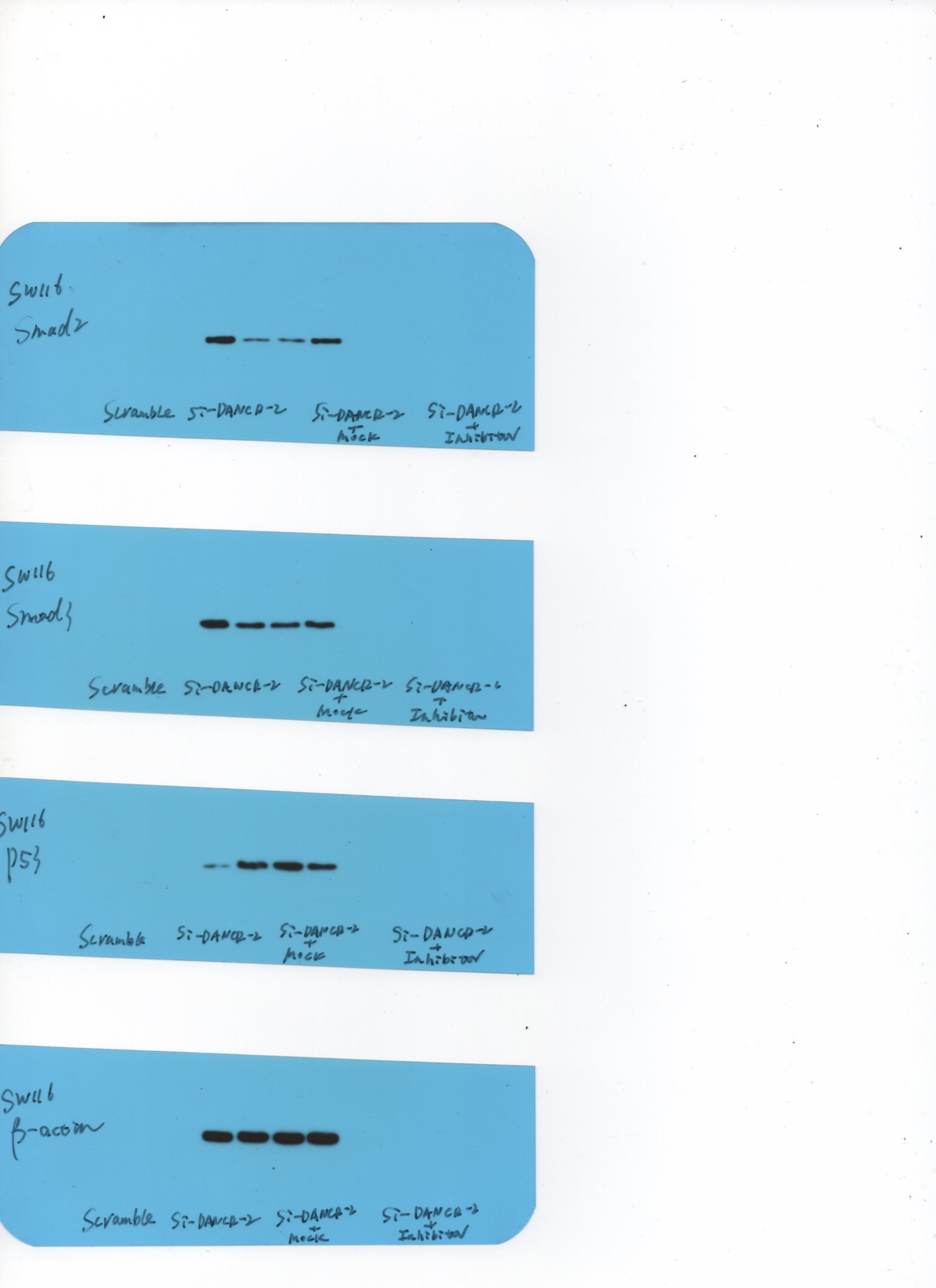

Supplement: Supplementary file 1 — Additional file 1: Figure S1. Original gels and blots of E-cadherin, Snail, Vimentin and β-actin (Corresponding to Fig. 2f in the manuscript). Figure S2. Original gels and blots of MDM2 and β-actin (Corresponding to Fig. 3i in the manuscript). Figure S3. Original gels and blots of E-cadherin, Snail, Vimentin and β-actin (Corresponding to Fig. 4f in manuscript). Figure S4. Original gels and blots of Smad2, Smad3, p53and β-actin of H29 and SW116 cells (Corresponding to Fig. 6 in manuscript). [file 12885_2020_6856_MOESM1_ESM.docx]
